# Supplementary figures and images for: High-throughput RNA sequencing from paired lesional- and non-lesional skin reveals major alterations in the psoriasis circRNAome
Source: BMC Med Genomics. 2019 Nov 27;12:174. doi: 10.1186/s12920-019-0616-2 (PMC6882360; doi:10.1186/s12920-019-0616-2)

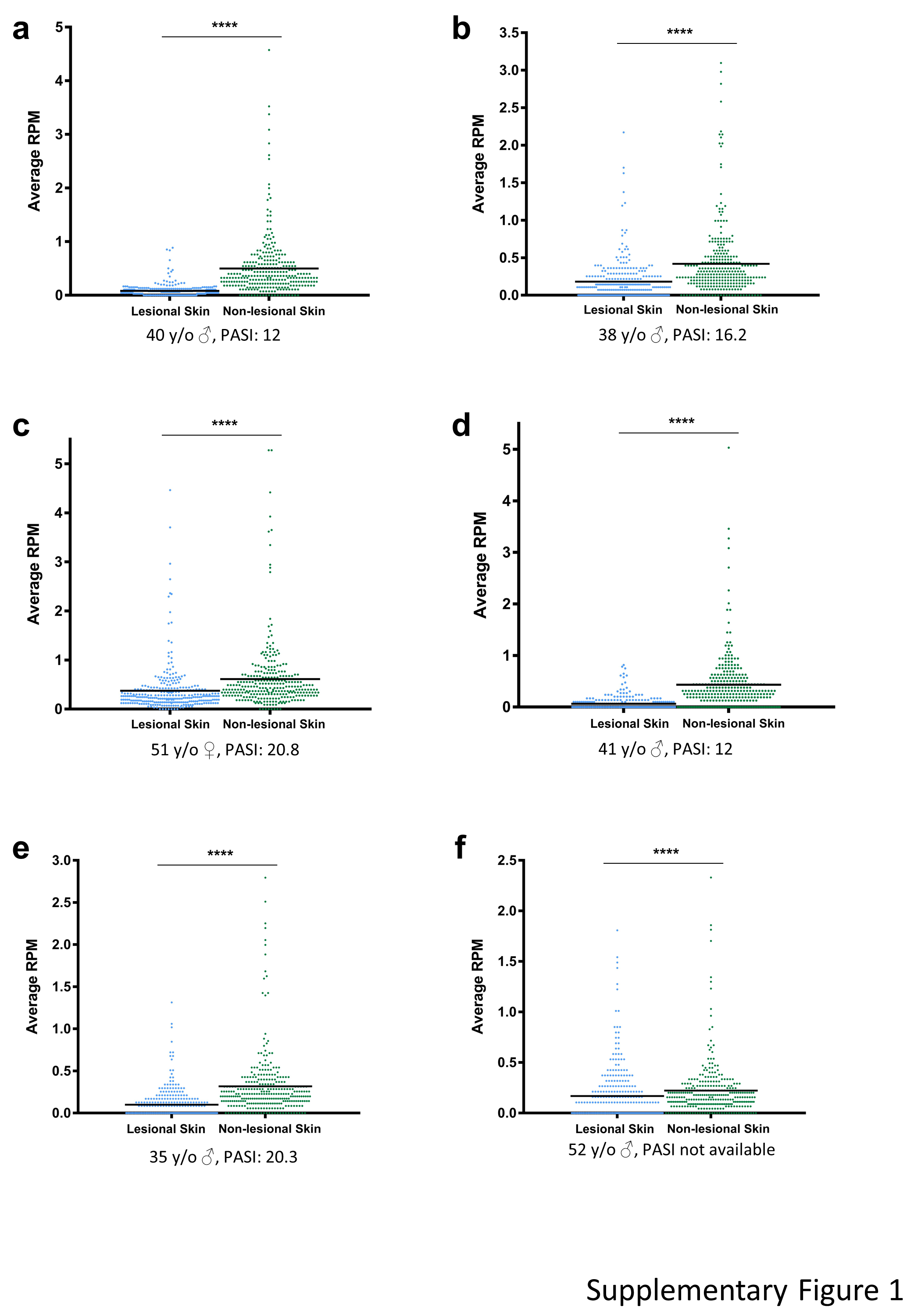

Supplement: Supplementary file 1 — Additional file 1: Figure S1. (a-f) Scatter plots showing the average expression in reads per million (RPM) of the 298 unique high-abundance circRNAs in lesional- and non-lesional skin for each of the individual patients. The bars represent means. [file 12920_2019_616_MOESM1_ESM.tif]

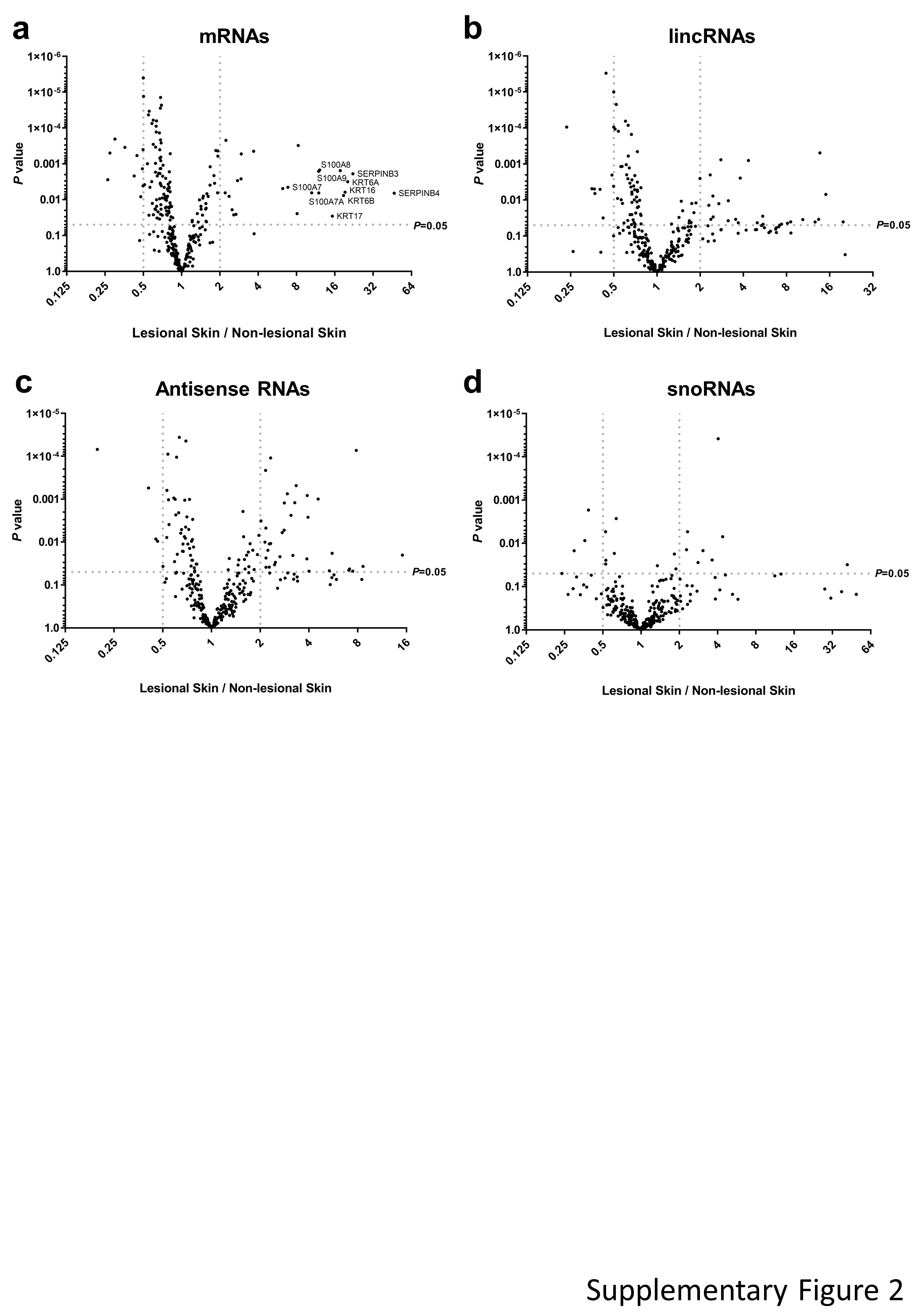

Supplement: Supplementary file 2 — Additional file 2: Figure S2. (a) Volcano plot of the top 298 most abundant mRNAs showing fold changes in mRNA expression between lesional- and non-lesional skin according to the levels of significance. Several genes known to be upregulated in psoriasis are indicated. (b) Volcano plot of the top 298 most abundant lincRNAs showing fold changes in lincRNA expression between lesional- and non-lesional skin according to the levels of significance. (c) Volcano plot of the top 298 most abundant antisense RNAs showing fold changes in antisense RNA expression between lesional- and non-lesional skin according to the levels of significance. (d) Volcano plot of the top 298 most abundant snoRNAs showing fold changes in snoRNA expression between lesional- and non-lesional skin according to the levels of significance. [file 12920_2019_616_MOESM2_ESM.tif]

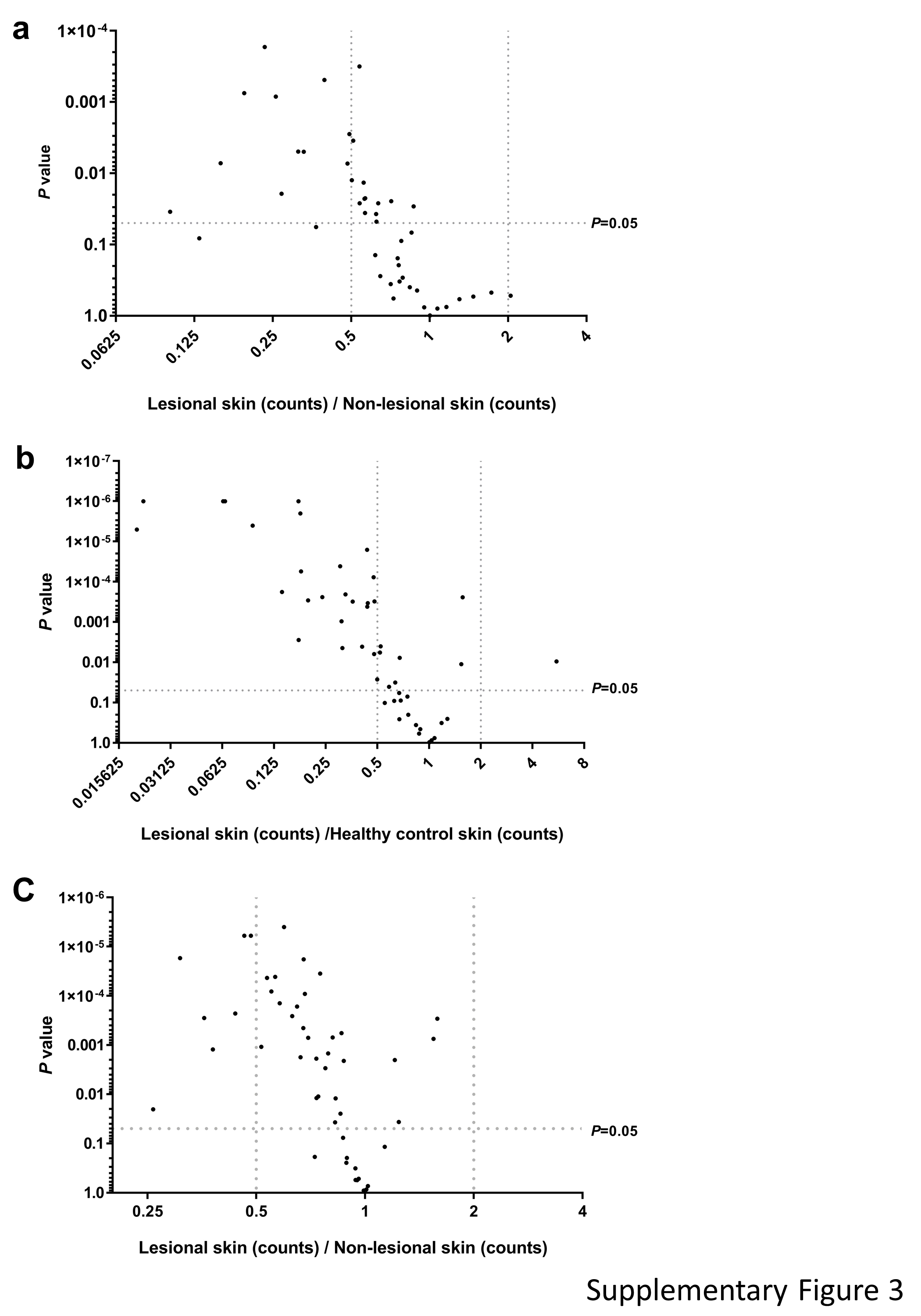

Supplement: Supplementary file 3 — Additional file 3: Figure S3. (a) Volcano plot of the top 50 most abundant circRNAs showing fold changes in circRNA expression in counts between lesional- and non-lesional skin from the first cohort according to the levels of significance. (b) Volcano plot of the top 50 most abundant circRNAs showing fold changes in circRNA expression in counts between lesional skin and healthy control skin according to the levels of significance. (c) Volcano plot of the top 50 most abundant circRNAs showing fold changes in circRNA expression in counts between lesional- and non-lesional skin from a second cohort of 13 patients according to the levels of significance. All analyses were performed using our custom NanoString nCounter panel. [file 12920_2019_616_MOESM3_ESM.tif]

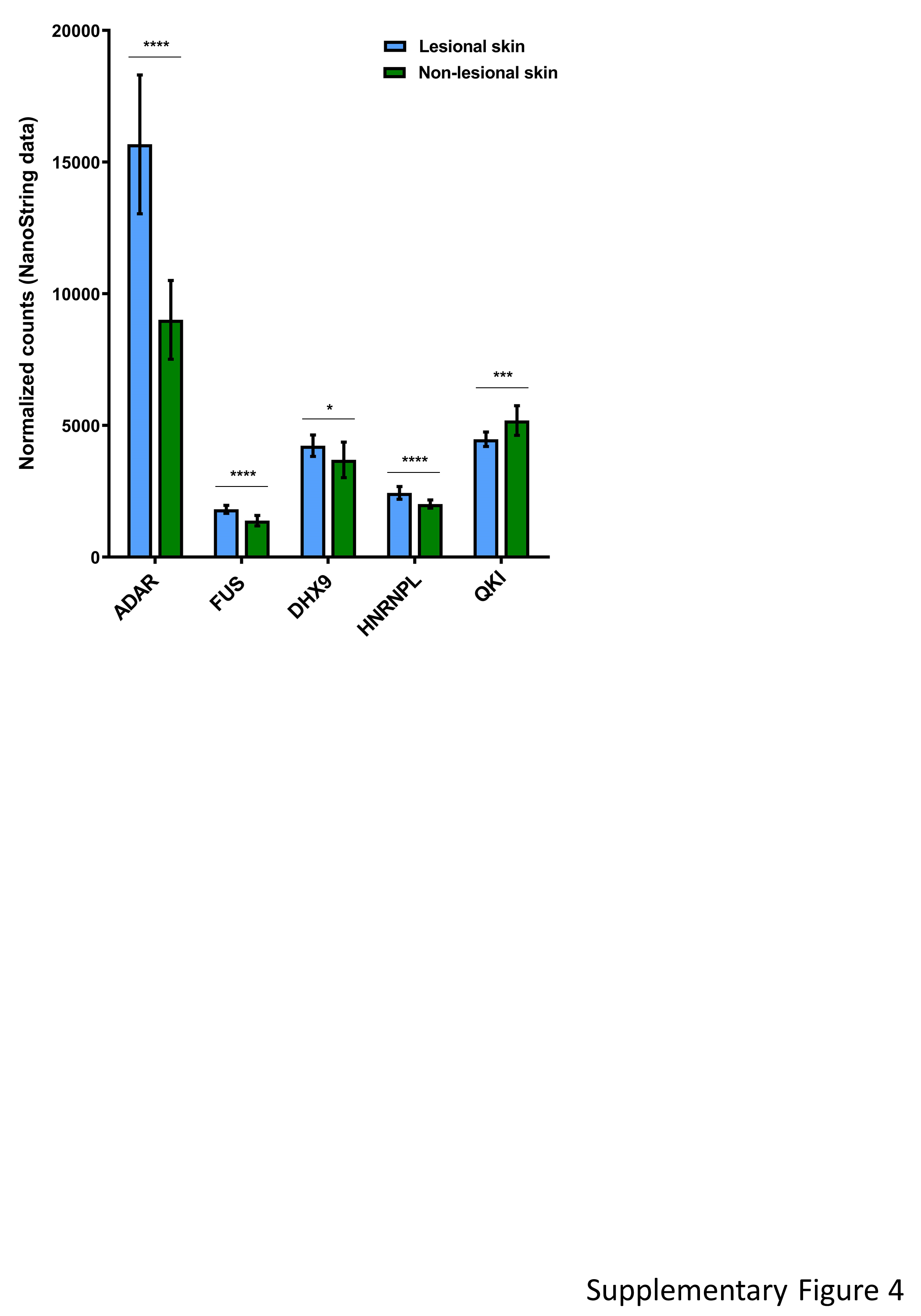

Supplement: Supplementary file 4 — Additional file 4: Figure S4. NanoString nCounter analysis confirmed that ADAR is upregulated in lesional- relative to non-lesional skin samples from a second cohort of 13 patients. [file 12920_2019_616_MOESM4_ESM.tif]

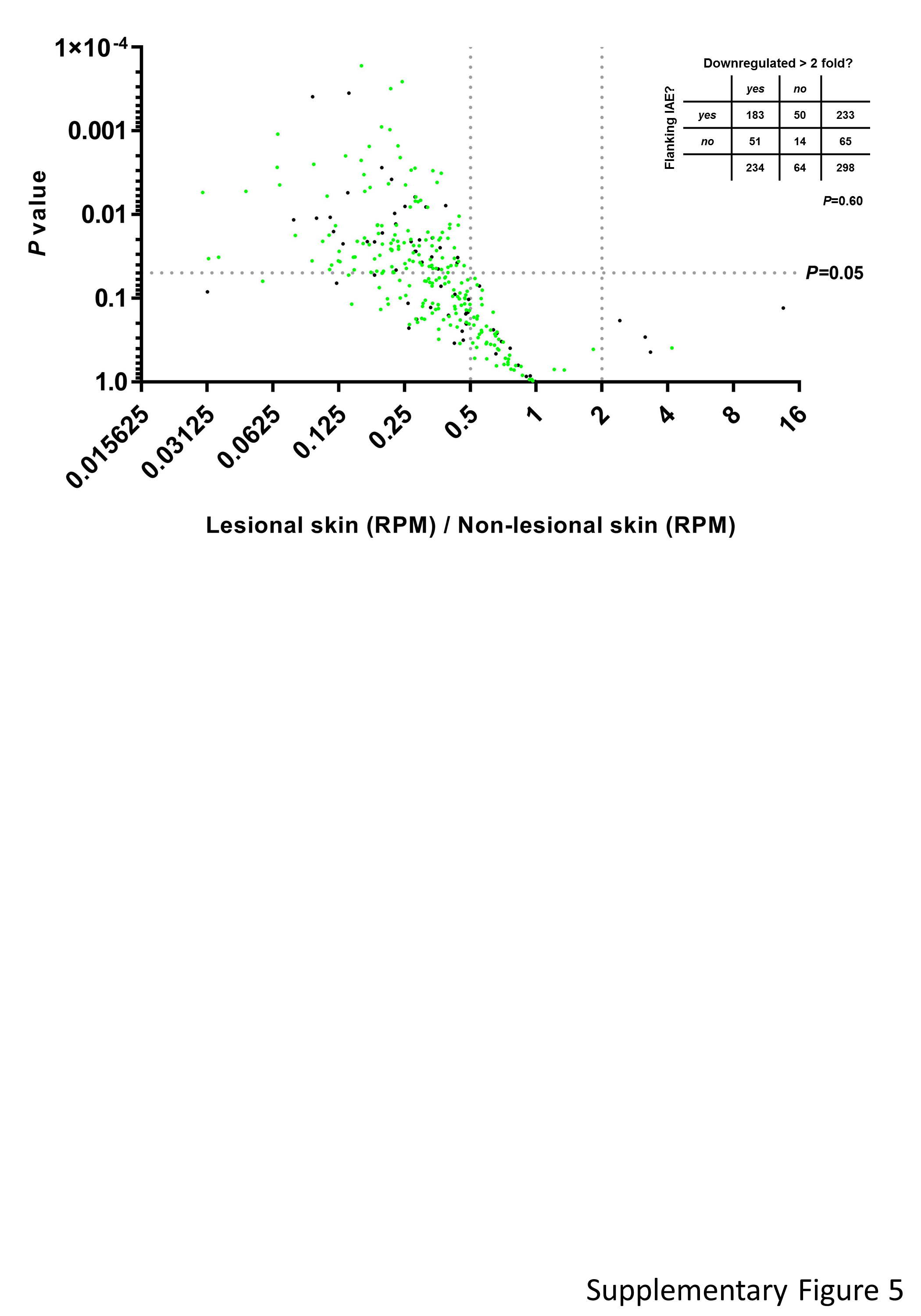

Supplement: Supplementary file 5 — Additional file 5: Figure S5. Volcano plot of the 298 unique high-abundance circRNAs showing fold changes in circRNA expression in RPM between lesional- and non-lesional skin according to the levels of significance. Two-hundred thirty-three circRNAs likely to have Alu-mediated biogenesis are indicated in green (flanked by IAEs from the same subfamily within 10,000 nucleotide regions flanking the BSJs). Numbers of circRNA in each category are shown in the inserted table. There was no statistically significant association between downregulation of circRNAs (more than 2 fold) and the presence of flanking IAEs from the same subfamily (P = 0.60, chi-squared test).) [file 12920_2019_616_MOESM5_ESM.tif]

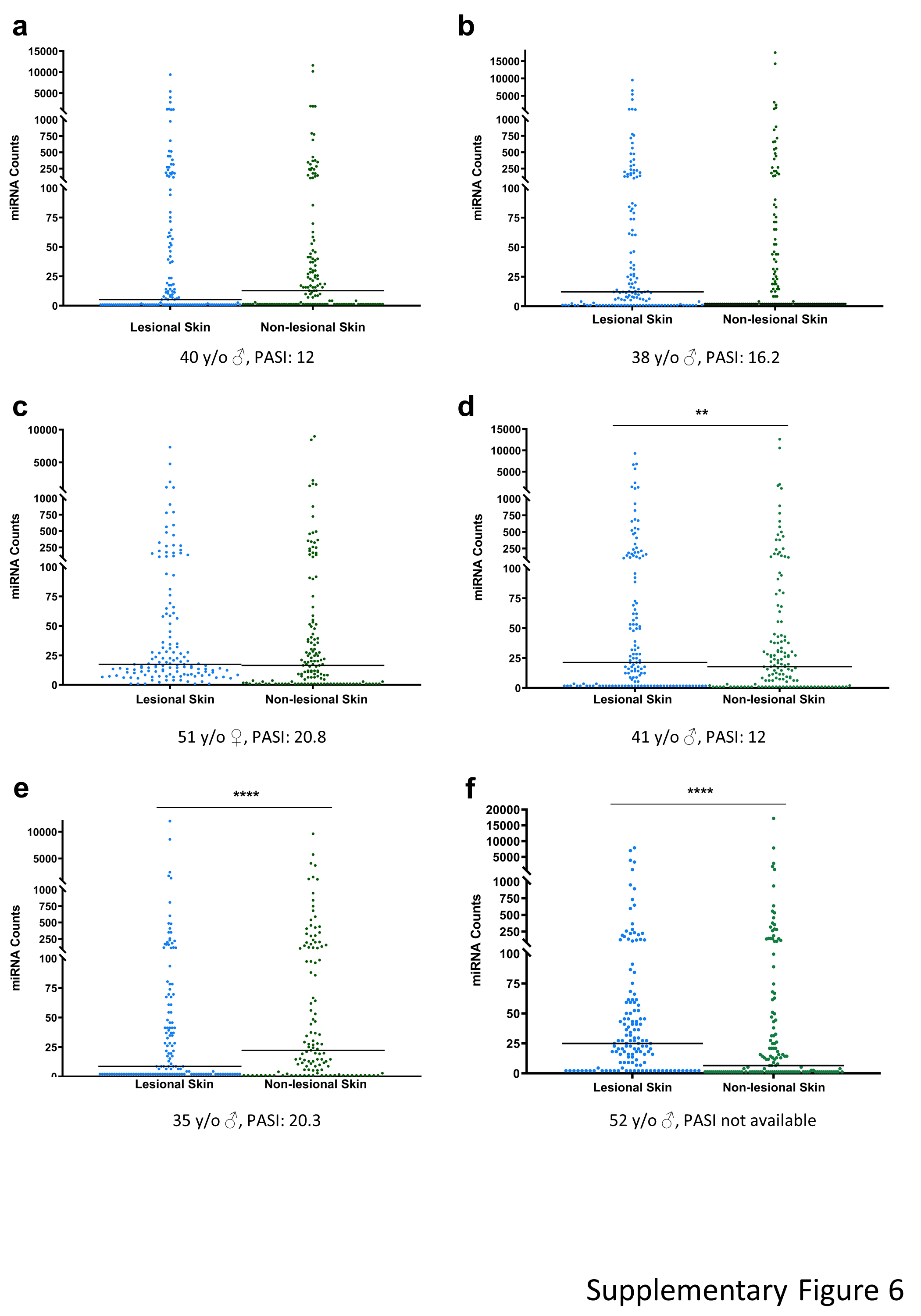

Supplement: Supplementary file 6 — Additional file 6: Figure S6. (a-f) Scatter plots showing the average expression in counts of the 137 unique high-abundance miRNAs in lesional- and non-lesional skin for each of the individual patients. The bars represent means. [file 12920_2019_616_MOESM6_ESM.tif]
